# Thromboprophylaxis and Risk Factors for Venous Thromboembolism in Critically Ill Children: A Hospital-Based Case-Control Study

**DOI:** 10.7759/cureus.91324

**Published:** 2025-08-31

**Authors:** Chimaobi Ezekiel Ijioma, Nelson Obioma Uzor, Oladoyin Ogunbayo Jolaoye, Uzoma Ndukwe, Ihechiluru Lazarus Nwokeafor, Christopher C Okafor, Joy Enyioma Ben, Emmanuel Akabueze, Wisdom O Ngumoha

**Affiliations:** 1 Pediatrics, Abia State Children's Specialist Hospital, Umuahia, NGA; 2 Pediatrics, University of Arkansas for Medical Sciences, Little Rock, USA; 3 Pediatrics, University Hospital, Southampton, GBR; 4 Internal Medicine-Pediatrics, University of Illinois College of Medicine at Peoria. OSF Medical Center, Peoria, USA; 5 Internal Medicine, Nigerian Christian Hospital, Onicha Ngwa, NGA; 6 Internal Medicine, Ebonyi State University Teaching Hospital, Abakaliki, NGA; 7 Family Medicine, 68 Nigerian Army Reference Hospital, Yaba, NGA; 8 Pediatrics, Abia State University Teaching Hospital, Aba, NGA

**Keywords:** case-control study, pediatric intensive care, risk factors, thromboprophylaxis, venous thromboembolism

## Abstract

Background and objective

Critically ill children in pediatric ICUs (PICUs) are at an elevated risk of venous thromboembolism (VTE) due to factors such as central venous catheterization, immobility, and underlying conditions. While thromboprophylaxis is well-established in adult critical care, its use in pediatric populations, particularly in resource-limited settings like Nigeria, remains under-researched and inconsistently applied. This study aimed to evaluate the use, effectiveness, and clinical outcomes of thromboprophylaxis in critically ill pediatric patients and to identify independent predictors of thromboembolic events.

Methods

A hospital-based case-control study was conducted at Abia State Children’s Specialist Hospital, Umuahia, Nigeria, from June 2023 to May 2025. A total of 180 critically ill children aged one month to 18 years were enrolled, comprising 60 cases with imaging-confirmed VTE and 120 age- and sex-matched controls without VTE. Data were collected using a structured case report form from medical records, laboratory and radiological reports, and direct clinical observation. Variables assessed included thromboprophylaxis type (mechanical or pharmacologic), laboratory parameters, and clinical outcomes. Multivariate logistic regression was used to identify independent predictors of VTE.

Results

Key risk factors for VTE included the presence of central venous catheters (adjusted odds ratio [AOR] = 2.84; p = 0.005), immobility for more than 48 hours (AOR = 2.76; p = 0.005), and BMI above the 95th percentile (AOR = 2.91; p = 0.035). Lack of thromboprophylaxis significantly increased the risk of VTE (AOR = 3.64; p<0.001). Both pharmacologic (p = 0.006) and combined pharmacologic-mechanical (p = 0.011) prophylaxis were associated with significantly reduced odds of VTE compared to no prophylaxis. Children with VTE had longer ICU stays (p<0.001), higher mortality rates (p = 0.010), and were more likely to present with abnormal coagulation profiles and elevated D-dimer levels.

Conclusions

Thromboprophylaxis, particularly pharmacologic or combined approaches, appears to be effective in reducing VTE among critically ill pediatric patients. However, its utilization remains suboptimal. The implementation of risk-based prophylaxis protocols and early screening strategies is essential to improving outcomes in pediatric critical care, especially in resource-limited settings.

## Introduction

Venous thromboembolism (VTE), comprising deep vein thrombosis (DVT) and pulmonary embolism (PE), has traditionally been considered rare in children. However, emerging evidence indicates that critically ill pediatric patients, particularly those in ICUs, are at significantly increased risk due to multiple overlapping factors, including immobility, central venous catheter (CVC) use, mechanical ventilation, and systemic inflammatory responses [1]. A comprehensive narrative review underscores that hospitalization and critical illness markedly elevate VTE risk in children, although reported incidence rates vary widely depending on study methodology, patient comorbidities, and clinical settings [2].

Despite the recognition of these risks, the use of thromboprophylaxis in pediatric ICUs (PICUs) remains inconsistent and generally underutilized. For instance, a 2014 multinational study involving 59 PICUs across several countries found that although 87% of children had at least one risk factor for thrombosis, only 12.4% received pharmacologic prophylaxis despite 34.7% having formal indications based on existing consensus guidelines [3]. Similar patterns have been observed in regional studies from Spain and Portugal, where pharmacologic prophylaxis was underused, mechanical prophylaxis was rare, and standardized institutional protocols were lacking [4].

This gap in clinical practice is compounded by a scarcity of pediatric-specific data to inform thromboprophylaxis guidelines. Current practices often rely on extrapolation from adult studies, due to the limited availability of randomized controlled trials in children. Pediatric-related evidence for the effectiveness of mechanical prophylactic devices and anticoagulants remains sparse, contributing to considerable variability in prophylactic approaches across institutions [1]. Furthermore, although risk stratification tools exist, few have been validated across diverse PICU populations, highlighting the urgent need for context-sensitive protocols.

The coronavirus 2019 (COVID-19) pandemic further underscored both the necessity and complexity of thromboprophylaxis in pediatric critical care. Pediatric patients with severe COVID-19 or multisystem inflammatory syndrome in children (MIS-C) often exhibit coagulopathy and an elevated risk of VTE. In response, several consensus guidelines have recommended prophylaxis with low-molecular-weight heparin (LMWH) or unfractionated heparin in children with markedly elevated D-dimer levels or other clinical risk factors [5]. One study evaluating pharmacologic thromboprophylaxis in pediatric patients with COVID-19/MIS-C found it feasible but emphasized the importance of multidisciplinary risk assessment and individualized management strategies [6].

In Nigeria and across much of Sub-Saharan Africa, there is a notable dearth of data on pediatric thromboprophylaxis. Existing literature is predominantly derived from high-income countries, where clinical resources and patient demographics differ significantly from those in low- and middle-income regions. The absence of context-specific empirical data presents a significant challenge to clinicians at institutions such as Abia State Children’s Specialist Hospital in Umuahia, who must make critical care decisions in the absence of localized evidence.

Given the global underuse of thromboprophylaxis, the lack of standardized pediatric protocols, and the scarcity of data from resource-limited settings, this study aims to address an important gap. The primary objective of this study was to evaluate thromboprophylaxis practices and associated outcomes among critically ill children in Umuahia. The secondary objectives were to identify risk factors for VTE in this population, to inform the development of evidence-based and context-appropriate protocols for pediatric VTE prevention in resource-limited settings, and to contribute to the broader understanding of pediatric VTE prevention in variable-resource environments.

## Materials and methods

Study design

This hospital-based prospective case-control study was conducted to determine the risk factors associated with venous VTE and to assess the utilization and effectiveness of thromboprophylaxis among critically ill children admitted to the PICU. The study also evaluated the prevalence of thromboprophylaxis use and compared clinical outcomes, including VTE occurrence and mortality, between patients who received prophylaxis and those who did not.

Study setting

The study was conducted between June 2023 and May 2025 at Abia State Children’s Specialist Hospital, Umuahia, a pediatric referral facility that provides outpatient, inpatient, intensive care, immunization, nutritional counseling, and follow-up services for children under 18 years. The PICU has 35 beds and admits approximately 300 critically ill children annually. The hospital caters to both urban and rural communities in and around Umuahia, the capital of Abia State, Nigeria.

Study participants and eligibility criteria

The study population included critically ill children aged one month to 18 years admitted to the PICU during the study period. Eligible participants had at least one predefined risk factor for VTE based on modified pediatric thrombosis criteria.

Inclusion criteria

Inclusion criteria comprised children aged one month to 18 years admitted to the PICU who presented with at least one of the following VTE risk factors: immobility exceeding 48 hours (defined as no ambulation or repositioning documented in nursing notes), presence of a CVC, sepsis (defined by established clinical and laboratory criteria), malignancy, congenital heart disease, obesity (BMI above the 95th percentile based on CDC growth charts), or a history of thromboembolism.

Exclusion criteria

Children were excluded if they had active bleeding or known bleeding disorders, were already on therapeutic anticoagulation for pre-existing thrombotic conditions, had incomplete clinical records, or if their parents/guardians withdrew consent.

Thromboprophylaxis protocol

Thromboprophylaxis was administered according to the clinician's discretion and local protocols. Pharmacologic prophylaxis (e.g., LMWH or unfractionated heparin) was used selectively for high-risk patients, while mechanical prophylaxis (e.g., compression stockings or intermittent pneumatic compression devices) was rarely applied due to resource constraints. Controls included PICU patients without evidence of VTE who may or may not have received thromboprophylaxis.

Sample size determination

Sample size was calculated using Epi Info™ version 7 as described by Kelsey et al. [7], based on the assumption of a 30% exposure rate to thromboprophylaxis among controls, odds ratio [OR] of 2.0, 95% confidence level, 80% power, and a case-control ratio of 1:2. A minimum of 60 cases (children who developed thromboembolism) and 120 controls (critically ill children without thromboembolism) were included, resulting in a total sample size of 180 participants. The formula used for the calculation is as follows:

$n_1 = \frac{\left( Z_{1-\alpha/2} \sqrt{r p_0 q_0 + p_1 q_1} + Z_{1-\beta} \sqrt{\frac{r p_0 q_0 + p_1 q_1}{r}} \right)^2}{(\Delta p)^2 r}$

$n_2 = r n_1$

In this approach, n_1_ represents the number of cases, n_2_ represents the number of controls, P_0_ is the estimated proportion of controls exposed (e.g., 0.30), and q_0 _= 1 − p_0_​​. The proportion of cases exposed, p_1_, is calculated using the formula 

$p_1 = \frac{OR \times p_0}{1 + p_0 (OR - 1)}$

Where OR is the anticipated odds ratio of exposure between cases and controls. Similarly, q_1_ = 1− p_1_, and the difference in proportions is denoted as Δp = p_1_− p_0_. The control-to-case ratio is represented by r (2:1). Z_1_−α_/2_​ corresponds to the standard normal deviate for a two-sided significance level (approximately 1.96 for α = 0.05), while Z_1_−β corresponds to the desired power of the study (approximately 0.84 for 80% power). These values were used to compute the minimum sample size required to detect a statistically significant association between exposure and outcome. The 30% exposure rate was estimated based on findings from previous multicenter studies reporting thromboprophylaxis rates among critically ill children [3].

Sampling technique

Cases were recruited consecutively from all critically ill children diagnosed with VTE during the study period, confirmed by imaging such as Doppler ultrasound or CT angiography. For each case, two age- and sex-matched controls (within ±1 year of age) without clinical or radiological evidence of thromboembolism were selected from PICU admissions.

Data collection procedures

Data were collected using a structured case report form (CRF) developed by the authors, and the CRF was developed from various studies (Appendix) [3,8-15]. Data collectors received standardized training to ensure uniform abstraction. Information was obtained from medical records (admission notes, nursing charts, and drug administration sheets), direct clinical observation, and laboratory/imaging reports (platelet count, prothrombin time, activated partial thromboplastin time [aPTT], and D-dimer). The CRF captured demographic variables (age, sex, weight, height, and BMI percentile), clinical characteristics (immobilization duration, presence of CVC, sepsis, malignancy, congenital heart disease, and previous VTE), thromboprophylaxis type, and clinical outcomes (VTE occurrence, bleeding complications, ICU length of stay, and survival).

Case and control definitions

The cases included critically ill children who developed objectively confirmed VTE (DVT or pulmonary embolism) diagnosed by Doppler ultrasound, CT angiography, or autopsy. Controls were critically ill children admitted during the same period without evidence of VTE.

Quality control and bias minimization

To reduce information bias, VTE diagnoses were confirmed by two independent radiologists blinded to thromboprophylaxis status. Data quality was assured through double data entry and periodic cross-checking.

Data analysis

Data were analyzed using IBM SPSS Statistics for Windows, Version 20 (IBM Corp., Armonk, NY). Continuous variables were summarized with means and standard deviations (SD), and categorical variables with frequencies and percentages. Bivariate analyses employed chi-square tests for categorical variables and Student’s t-tests for continuous variables. Variables with p-values <0.20 in bivariate analysis were included in a multivariate logistic regression model to identify independent predictors of VTE. Statistical significance was set at p<0.05.

Ethical considerations

Ethical approval for the study was obtained from the Ethics Committee of Abia State Children's Specialist Hospital, Umuahia, Nigeria, with approval number ABSCSH/EC/23/051, dated May 20, 2023. Written informed consent was obtained from parents or guardians, and assent was obtained from children aged seven years and older via an age-appropriate process. Data confidentiality was maintained by storing records in a password-protected electronic database accessible only to authorized personnel. Participation was voluntary, with the option to withdraw at any time without affecting clinical care.

## Results

A total of 180 children were included in the study: 60 cases and 120 controls. The mean age of cases was 6.6 ± 4.2 years, compared to 6.3 ± 4.1 years among controls (t = 0.63, p = 0.532). There was no significant difference in sex distribution between the groups, with 33 (55.0%) males among cases and 62 (51.7%) among controls (χ² = 0.17, p = 0.678). Children with BMI >95th percentile were 12 (20.0%) among cases and 10 (8.3%) among controls (χ² = 4.91, p = 0.027). Immobility >48 hours was observed in 42 (70.0%) cases and 54 (45.0%) controls (χ² = 9.68, p = 0.002). The presence of CVC was reported in 37 (61.7%) cases and 46 (38.3%) controls (χ² = 8.87, p = 0.003). A previous history of VTE was documented in six (10.0%) cases and two (1.7%) controls (χ² = 6.13, p = 0.013). Other variables such as sepsis were present in 25 (41.7%) cases and 34 (28.3%) controls (χ² = 3.10, p = 0.079); congenital heart disease in eight (13.3%) cases and 10 (8.3%) controls (χ² = 1.08, p = 0.300); and malignancy in five (8.3%) cases and six (5.0%) controls (χ² = 0.74, p = 0.390). These differences were not statistically significant (Table 1).

**Table 1:** Demographic and clinical characteristics of cases and controls An independent sample t-test (t) was used for the means of the continuous variable, and a chi-square (χ²) test was used to examine the relationship between the categorical variables. A p-value <0.05 was considered statistically significant BMI: body mass index; SD: standard deviation; VTE: venous thromboembolism

Variable	Cases (n = 60)	Controls (n = 120)	Total (N = 180)	Test statistic	P-value
Age, years, mean ± SD	6.6 ± 4.2	6.3 ± 4.1	6.4 ± 4.1	t = 0.63	0.532
Sex, Male, n (%)	33 (55.0%)	62 (51.7%)	95 (52.8%)	χ² = 0.17	0.678
BMI >95th percentile n (%)	12 (20.0%)	10 (8.3%)	22 (12.2%)	χ² = 4.91	0.027
Immobility >48 hours, n (%)	42 (70.0%)	54 (45.0%)	96 (53.3%)	χ² = 9.68	0.002
CVC in place, n (%)	37 (61.7%)	46 (38.3%)	83 (46.1%)	χ² = 8.87	0.003
Sepsis, n (%)	25 (41.7%)	34 (28.3%)	59 (32.8%)	χ² = 3.10	0.079
Congenital heart disease, n (%)	8 (13.3%)	10 (8.3%)	18 (10.0%)	χ² = 1.08	0.300
Malignancy, n (%)	5 (8.3%)	6 (5.0%)	11 (6.1%)	χ² = 0.74	0.390
Previous VTE history, n (%)	6 (10.0%)	2 (1.7%)	8 (4.4%)	χ² = 6.13	0.013

As seen in Table 2, among the 60 cases, 35 (58.3%) received no thromboprophylaxis compared to 40 (33.3%) controls. Mechanical thromboprophylaxis alone was used in nine (15.0%) cases and 17 (14.2%) controls, with an odds ratio (OR) of 0.60 (95% CI: 0.2-1.5), which was not statistically significant (Wald χ² = 1.20, p = 0.270). Pharmacologic thromboprophylaxis was administered to 10 (16.7%) cases and 37 (30.8%) controls, showing a significant protective association with 69% reduced odds of VTE (OR = 0.31; 95% CI: 0.1-0.7; Wald χ² = 7.52, p = 0.006). Combined mechanical and pharmacologic thromboprophylaxis was used in 6 (10.0%) cases and 26 (21.7%) controls, also demonstrating a significant 74% reduction in the odds of VTE (OR = 0.26; 95% CI: 0.09-0.74; Wald χ² = 6.45, p = 0.011).

**Table 2:** Thromboprophylaxis characteristics A binary logistic regression was used to calculate the OR. Wald chi-square (χ²) test statistic values are reported. A p-value <0.05 was considered statistically significant CI: confidence interval; OR: odds ratios

Type of thromboprophylaxis	Cases, n (%) (n = 60)	Controls, n (%) (n = 120)	OR (95% CI)	Wald χ²	P-value
None	35 (58.3%)	40 (33.3%)	Reference	—	—
Mechanical only	9 (15.0%)	17 (14.2%)	0.60 (0.2–1.5)	1.20	0.270
Pharmacologic only	10 (16.7%)	37 (30.8%)	0.31 (0.1–0.7)	7.52	0.006
Combined	6 (10.0%)	26 (21.7%)	0.26 (0.09–0.74)	6.45	0.011

As shown in Table 3, the mean platelet count was lower in cases (280.5 ± 95.2 × 10⁹/L) compared to controls (305.1 ± 91.4 × 10⁹/L), but this difference was not statistically significant (t = -1.71, p = 0.092). Cases had significantly prolonged prothrombin time (PT), with a mean of 17.2 ± 4.8 seconds versus 14.6 ± 3.2 seconds in controls (t = 3.83, p = 0.001). Similarly, aPTT was significantly longer among cases (43.6 ± 6.3 seconds) compared to controls (37.2 ± 5.4 seconds) (t = 6.58, p<0.001). D-dimer levels were also significantly elevated in cases (3.24 ± 1.12 mg/L) relative to controls (1.98 ± 0.84 mg/L) (t = 7.38, p<0.001).

**Table 3:** Laboratory findings An independent sample t-test was used for comparison of means. A p-value <0.05 was considered statistically significant aPTT: activated partial thromboplastin time; PT: prothrombin time; SD: standard deviation

Laboratory parameter	Cases, mean ± SD (n = 60)	Controls, mean ± SD (n = 120)	t-test	P-value
Platelet count, ×10⁹/L	280.5 ± 95.2	305.1 ± 91.4	-1.71	0.092
PT, seconds	17.2 ± 4.8	14.6 ± 3.2	3.83	0.001
aPTT, seconds	43.6 ± 6.3	37.2 ± 5.4	6.58	<0.001
D-dimer, mg/L	3.24 ± 1.12	1.98 ± 0.84	7.38	<0.001

The mean length of ICU stay was significantly longer for cases (14.2 ± 5.1 days) compared to controls (9.3 ± 4.2 days) (t = 6.62, p<0.001). Bleeding complications occurred in five (8.3%) cases and six (5.0%) controls, but this difference was not statistically significant (χ² = 0.81, p = 0.370). Mortality was significantly higher among cases, with 19 (31.7%) deaths compared to 18 (15.0%) deaths in controls (χ² = 6.58, p = 0.010) (Table 4).

**Table 4:** Clinical outcomes of cases and controls An independent sample t-test (t) was used for the means of the continuous variable, and a chi-square (χ²) test was used to examine the relationship between the categorical variables. A p-value <0.05 was considered statistically significant. ICU: intensive care unit; SD: standard deviation

Outcome	Cases (n = 60)	Controls (n = 120)	Test statistic	P-value
ICU stay, days, mean ± SD	14.2 ± 5.1	9.3 ± 4.2	t = 6.62	<0.001
Bleeding complications, n (%)	5 (8.3%)	6 (5.0%)	χ² = 0.81	0.370
Mortality, n (%)	19 (31.7%)	18 (15.0%)	χ² = 6.58	0.010

As seen in Table 5, multivariate logistic regression analysis identified several independent predictors of VTE among critically ill children. The presence of a CVC was associated with nearly threefold increased odds of VTE (adjusted odds ratio [AOR] = 2.84; 95% CI: 1.37-5.91; Wald χ² = 7.79, p = 0.005). Immobility for more than 48 hours similarly increased VTE risk (AOR = 2.76; 95% CI: 1.35-5.64; Wald χ² = 7.73, p = 0.005). Children with BMI above the 95th percentile had significantly higher odds of VTE (AOR = 2.91; 95% CI: 1.08-7.86; Wald χ² = 4.42, p = 0.035). Lack of thromboprophylaxis was the strongest predictor, associated with a more than threefold increase in VTE odds (AOR = 3.64; 95% CI: 1.74-7.63; Wald χ² = 11.82, p<0.001). Congenital heart disease was not a statistically significant predictor (AOR = 2.11; 95% CI: 0.80-5.40; Wald χ² = 2.34, p = 0.127).

**Table 5:** Multivariate logistic regression analysis of predictors of VTE A multivariable logistic regression was used to determine independent predictors of VTE. Wald Chi-square (χ²) test statistic values are reported. A p-value <0.05 was considered statistically significant. BMI: body mass index; CI: confidence interval; CVC: central venous catheter; VTE: venous thromboembolism

Predictor	Adjusted odds ratio (AOR)	95% CI	Wald χ²	P-value
CVC in place	2.84	1.37–5.91	7.79	0.005
Immobility >48 hours	2.76	1.35–5.64	7.73	0.005
BMI >95th percentile	2.91	1.08–7.86	4.42	0.035
Congenital heart disease	2.11	0.8–5.4	2.34	0.127
No thromboprophylaxis	3.64	1.74–7.63	11.82	<0.001

## Discussion

VTE is a significant and potentially life-threatening complication among critically ill pediatric patients, particularly those admitted to PICUs [1]. Despite its recognized risk, thromboprophylaxis, which entails the prevention of blood clots using mechanical or pharmacologic methods, is inconsistently applied in children, especially in resource-limited settings such as Nigeria [2]. The underuse of thromboprophylaxis contributes to higher rates of morbidity and mortality in this vulnerable population. Understanding the risk factors, patterns of thromboprophylaxis use, and clinical outcomes is essential for developing effective prevention strategies. This study evaluates the use, effectiveness, and outcomes of thromboprophylaxis among critically ill children at Abia State Children’s Specialist Hospital, Umuahia, Nigeria.

The study included 180 children, with 60 cases and 120 controls. The mean age was similar between cases (6.6 years) and controls (6.3 years). This study identified several significant factors associated with VTE in critically ill pediatric patients. Notably, obesity, as indicated by a BMI above the 95th percentile, was significantly more prevalent among cases (20.0%) compared to controls (8.3%). This finding aligns with existing literature demonstrating obesity as an independent risk factor. Obesity is associated with inactivity, raised intra-abdominal pressure, a chronic low-grade inflammatory state, impaired fibrinolysis, high levels of fibrinogen, von Willebrand factor, and factor VIII, leading to a prothrombotic condition and elevated risk of VTE [8]. It has been shown that the risk of VTE increases with increasing BMI, and the risk is higher when obesity interacts with other thrombotic risk factors [8].

Prolonged immobility (>48 hours) was another key risk factor significantly associated with VTE, occurring in 70.0% of cases versus 45.0% of controls. Immobility leads to venous stasis, a well-recognized component of Virchow’s triad, which predisposes patients to thrombus formation [9]. This is consistent with findings in critically ill children and adults, where immobilization remains one of the strongest predictors of thrombotic events [3]. The presence of a CVC was significantly higher among cases (61.7%) compared to controls (38.3%), representing a major VTE risk factor in pediatric intensive care. CVCs disrupt venous integrity and promote localized thrombus formation, especially when indwelling for prolonged periods [10]. Although sepsis was more common among cases (41.7%) than controls (28.3%), this association did not reach statistical significance in our study. Similarly, congenital heart disease and malignancy showed no significant difference between cases and controls, likely due to smaller sample sizes or differing thrombosis mechanisms. A prior history of VTE was reported in 10.0% of cases compared to 1.7% of controls, highlighting its role as a strong predictor for recurrence [11].

This study explored the relationship between the type of thromboprophylaxis administered and the occurrence of VTE in critically ill pediatric patients. A considerable proportion of cases (58.3%) did not receive any form of thromboprophylaxis, compared to 33.3% of controls. This finding underscores the heightened risk of VTE in children who do not receive preventive measures. The low overall use of thromboprophylaxis in pediatric ICU populations is a recurring theme. Faustino et al. (2014), in their multinational PICU practice study, found that despite 86.9% of children having ≥1 VTE risk factor, only 12.4% received pharmacologic prophylaxis [3]. This practice gap parallels our study. Clearly, there is an urgent need to deploy context-appropriate thromboprophylactic strategies.

In contrast, pharmacologic prophylaxis alone was more frequently administered among controls (30.8%) than cases (16.7%), suggesting a protective benefit. Combined prophylaxis (both mechanical and pharmacologic) was also more common in the control group (21.7%) compared to the case group (10.0%), indicating a potentially greater preventive effect when both methods are used together. Patients who received pharmacologic thromboprophylaxis only had a significantly reduced odds of VTE (69%), as did those who received combined prophylaxis (both pharmacologic and mechanical), which showed an even lower odds (74%). These patterns support current recommendations favoring pharmacologic or combined approaches in high-risk pediatric populations [9,12]. Mechanical prophylaxis alone was observed at nearly equal rates in both groups (15.0% in cases and 14.2% in controls), and it appeared to offer limited protection when used in isolation. This may be due to size-related challenges and compliance issues.

Our study revealed significant differences in coagulation profiles between children with VTE (cases) and those without (controls). Although the mean platelet count was lower in cases compared to controls, the difference was not statistically significant. This suggested that thrombocytopenia did not appear to be a key distinguishing factor in this cohort. However, we observed significantly prolonged prothrombin time and aPTT in the VTE group. These findings suggest a state of coagulation imbalance among the cases, possibly reflecting consumptive coagulopathy or underlying sepsis-related coagulopathies. D-dimer levels were also markedly elevated in the case group compared to the control group. Elevated D-dimer is a well-established marker of fibrinolysis and thrombus formation, and its diagnostic utility in pediatric VTE has been previously reported [13]. The significantly higher D-dimer levels observed in this study support its continued use as a biomarker for early detection and monitoring of thrombosis in critically ill pediatric patients.

Patients who developed VTE had a significantly longer stay in the ICU, with an average of 14.2 days compared to 9.3 days among controls. This extended length of stay may reflect the added burden of managing thrombotic complications and their sequelae. Similar findings have been reported in pediatric cohorts, where VTE was associated with prolonged hospitalization, increased healthcare utilization, and a greater likelihood of invasive interventions [14]. Bleeding complications occurred in 8.3% of cases and 5.0% of controls. Although not statistically significant, this difference may be clinically relevant in the context of thromboprophylaxis, especially in critically ill children who are simultaneously at risk for both thrombosis and bleeding. This presents a challenge in pediatric critical care between thrombosis prevention and bleeding risk.

Mortality was notably higher among cases, with 31.7% of children with VTE dying during admission compared to 15.0% of controls, portraying the severity of thromboembolic complications in pediatric critical care. This observation aligns with existing pediatric data: a large multicenter U.S. cohort study reported mortality of 13.4% in children with VTE versus 3.5% in children without VTE [15]. This reflects both the direct impact of thrombosis and the complexity of underlying comorbidities. Furthermore, a population-based research from Quebec found an all-cause mortality rate of approximately 11.4 per 1,000 child-years among children with VTE, higher than background age-specific mortality estimates, and consistent with registry data reporting pediatric VTE-related mortality rates between 9-17% [16]. Collectively, these findings indicate that pediatric VTE is associated with significantly elevated mortality and should be considered a critical factor in outcome prediction and clinical decision-making in PICUs.

Being in a low-resource setting means that patients often present to the hospital in a more advanced state of illness, having experienced these risk factors, such as immobility or infection at home, for extended periods before seeking care. This delay in presentation likely contributes to the higher rates of complications, including longer ICU stays and elevated mortality observed in this cohort. In such a setting, where demand for intensive care frequently exceeds available capacity, complications like VTE that prolong ICU stay have broader implications beyond individual morbidity and mortality. Prolonged admissions limit turnover, thereby reducing opportunities for other critically ill children to access intensive care services. This highlights the importance of prevention strategies. Although a formal cost-analysis was not performed, systematic implementation of VTE prophylaxis protocols may offer potential cost savings through reduced complications and shorter ICU stays, while simultaneously improving equity of access by optimizing use of limited PICU resources.

Multivariable logistic regression analysis identified several independent predictors of VTE among critically ill children. Notably, having a CVC in place was associated with nearly three times increased odds of developing VTE (58.3% in cases vs. 38.3% in controls). This finding is consistent with literature showing CVCs as the most significant modifiable risk factor for hospital-acquired VTE in children, often implicated in over 70-90% of cases [14]. Prolonged immobility (≥48 hours) was also significantly associated with VTE, with 70.0% of cases affected compared to 45.0% of controls. Immobility contributes to venous stasis, which is a leading event in Virchow’s triad [17]. Children with obesity (BMI >95th percentile) also had a significantly higher likelihood of VTE (20.0% in cases vs. 8.3% in controls). Obesity has been increasingly recognized as a prothrombotic condition in children due to chronic inflammation and impaired fibrinolysis [18].

Although congenital heart disease was more common in cases (13.3%) than controls (8.3%), it did not reach statistical significance in this study. Importantly, the absence of thromboprophylaxis was strongly associated with VTE risk, with 58.3% of cases having received no prophylaxis versus 33.3% of controls. This underscores the importance of timely and appropriate thromboprophylaxis, particularly in resource-limited settings where delays in intervention may contribute to worse outcomes. Innovative data from a large registry show that critically ill pediatric patients not receiving thromboprophylaxis had significantly higher rates of hospital-acquired VTE compared to those who did receive it. In one study, hospital-wide implementation of a risk-stratified protocol including explicit prophylaxis guidelines was associated with a 33% reduction in VTE incidence, strongly suggesting that the lack of prophylaxis contributes directly to increased VTE risk [19].

Recommendations

Implement Routine VTE Risk Assessment Protocols

All hospitalized children, especially those in ICUs, should undergo systematic risk assessment for VTE using validated pediatric risk models to guide prophylactic decisions.

Strengthen Use of Thromboprophylaxis

Both pharmacologic and mechanical prophylaxis should be used appropriately in children identified as high-risk. Protocols should be tailored to balance thrombosis risk and bleeding concerns, especially in resource-limited settings.

Prioritize Early Mobilization Strategies

Prolonged immobility (>48 hours) significantly increases VTE risk. Hospitals should integrate physical therapy and early mobilization into pediatric critical care plans where feasible.

Improve Central Venous Catheter Management

Given the strong association between CVC use and VTE, there should be stricter criteria for catheter placement, daily review of necessity, and use of safer insertion techniques.

Address Obesity in Pediatric Populations

Obesity (BMI >95th percentile) is a modifiable risk factor. Public health programs should prioritize early identification and management of childhood obesity to reduce both short- and long-term thrombotic risks.

Enhance Early Detection and Management in Low-Resource Settings

Given the delayed presentation of critically ill children in low-resource areas, there should be community-based education on recognizing danger signs and seeking early care. In-hospital triage systems should be strengthened to rapidly identify and manage at-risk children.

Establish Institutional Protocols and Training

Facilities should develop institutional guidelines for pediatric VTE prevention and train healthcare providers on the recognition, prevention, and management of thromboembolic events.

Invest in Laboratory Infrastructure

To support evidence-based diagnosis and monitoring (e.g., D-dimer, prothrombin time, aPTT), hospitals must improve access to affordable and reliable coagulation assays in pediatric settings.

Encourage Further Research and Surveillance

Multicenter and longitudinal studies should be encouraged to explore the long-term outcomes of pediatric VTE, as well as the effectiveness of different prophylactic regimens across diverse populations.

Limitations

This study has several limitations. First, it was conducted in a single tertiary hospital, which limits the generalizability of the findings to other settings with different patient populations or resource availability. Secondly, the retrospective case-control design is inherently subject to biases such as incomplete or inaccurate documentation and potential misclassification of variables. The sample size, although sufficient to detect some significant associations, may not have been large enough to assess less common risk factors or outcomes such as congenital anomalies or malignancies. Additionally, unmeasured confounders, including genetic predisposition to thrombosis, socioeconomic status, and severity of illness scores, were not captured, which may have influenced the associations observed. Also, the study did not explore the dosing details or timing of prophylaxis, areas ripe for further investigation. The study also focused exclusively on in-hospital outcomes, without evaluating long-term complications such as recurrent VTE or post-thrombotic syndrome. Given the limited diagnostic resources available in a low-resource setting, it is possible that some cases of VTE were underdiagnosed or missed entirely. Severity-of-illness scores such as PRISM, PIM, or PELOD were not available, which may limit comparability with other PICU studies.

## Conclusions

This case-control study highlights the significant burden and associated risk factors of VTE among critically ill children in a low-resource tertiary care setting. In critically ill children admitted to Abia State Children’s Specialist Hospital, CVC placement, prolonged immobility, obesity, and lack of thromboprophylaxis independently predicted VTE, which was associated with worse clinical outcomes, including longer ICU stays and increased mortality. Pharmacologic and combined thromboprophylaxis significantly reduced the risk of VTE without increasing bleeding complications. These findings strongly support considering the implementation of risk-based thromboprophylaxis in Nigerian PICUs and conducting further studies to optimize strategies in resource-limited settings.

## Appendices

**Table 6:** Case Report Form: Thromboprophylaxis and Risk Factors for Venous Thromboembolism in Critically Ill Children: A Hospital-Based Case-Control Study Credits: Ijioma CE, Uzor NO, Jolaoye OO, Ndukwe U, Nwokeafor IL, Okafor CC, Ekweozor CJ, Ben JE, Akabueze E, Ngumoha WO

Section	Variable	Response format
Introduction & Consent	Dear Respondent, We are conducting a study titled “Thromboprophylaxis for Critically Ill Children: A Case-Control Study” at Abia State Children's Specialist Hospital, Umuahia. This research aims to assess the risk factors, use, and outcomes of thromboprophylaxis in children admitted to the intensive care unit (ICU). The information you provide will contribute significantly to improving pediatric care and outcomes. Your participation is voluntary, and all responses will be treated with strict confidentiality. You may withdraw from the study at any point without penalty. This study has received ethical approval from the relevant research ethics committee. Thank you for your time and cooperation.	
Informed Consent	Understand the nature and purpose	☐ Yes ☐ No
	Agree to participate	☐ Yes ☐ No
	Confidentiality assured	☐ Yes ☐ No
	Signature of respondent (or proxy)	____________________________
	Date	___ / ___ / ______
Section A: Demographics	Study ID	__________________
	Date of Admission	____ / ____ / 202___
	Age (years)	___________
	Sex	☐ Male ☐ Female
	Weight (kg)	___________
	Height (cm)	___________
	BMI Percentile	☐ < 95th percentile ☐ > 95th percentile
Section B: Clinical Characteristics	Immobility > 48 hrs	☐ Yes ☐ No
	Central Venous Catheter (CVC)	☐ Yes ☐ No
	Diagnosis of Sepsis	☐ Yes ☐ No
	Congenital Heart Disease	☐ Yes ☐ No
	Malignancy	☐ Yes ☐ No
	Previous VTE History	☐ Yes ☐ No
Section C: Thromboprophylaxis Use	Thromboprophylaxis Type	☐ None ☐ Mechanical only ☐ Pharmacologic only ☐ Combined
Section D: Laboratory Parameters	Platelet Count (×10⁹/L)	___________
	Prothrombin Time (PT, sec)	___________
	aPTT (sec)	___________
	D-dimer (ng/mL or μg/mL FEU)	___________
Section E: Clinical Outcomes	Development of VTE	Yes ☐ No
	Duration of ICU Stay (days)	___________
	Bleeding Complications	☐ Yes ☐ No
	Outcome of Admission	☐ Survived ☐ Died
Conclusion	Thank you for your participation. Your cooperation is vital in helping us improve the clinical care of critically ill children, especially regarding the safe and effective use of thromboprophylaxis. If you have any questions about this study, feel free to contact the principal investigator.	
